# A conserved glycan motif induces broadly reactive functional antibodies against the zoonotic pathogen *Streptococcus suis*

**DOI:** 10.1126/sciadv.adz1854

**Published:** 2026-03-25

**Authors:** Yao Shi, Göran Widmalm, Charlotte Sorieul, Thomas J. Roodsant, Jeffrey S. Rush, Natalia Korotkova, Manouk Vrieling, Antonius A. C. Jacobs, Mirlin Spaninks, Ries Grommen, C. Coral Domínguez-Medina, Irene M. Schimmel, Nicole N. van der Wel, Cameron W. Kenner, Christian Heiss, Parastoo Azadi, Li Tan, Jeroen D. C. Codée, Arjan Stegeman, Constance Schultsz, Lindert Benedictus, Nina M. van Sorge

**Affiliations:** ^1^ Department of Population Health Sciences, Division Farm Animal Health, Utrecht University, Utrecht, Netherlands.; ^2^ Department of Medical Microbiology and Infection Prevention, Amsterdam UMC Location University of Amsterdam, Amsterdam, Netherlands.; ^3^ Department of Chemistry, Arrhenius Laboratory, Stockholm University, Stockholm, Sweden.; ^4^ Leiden Institute of Chemistry, Leiden University, Leiden, Netherlands.; ^5^ Department of Molecular and Cellular Biochemistry, University of Kentucky, Lexington, KY, USA.; ^6^ Department of Microbiology, Immunology and Molecular Genetics, University of Kentucky, Lexington, KY, USA.; ^7^ Wageningen Bioveterinary Research, Houtribweg 39, Lelystad, Netherlands.; ^8^ MSD Animal Health, Wim de Körverstraat 35, Boxmeer, Netherlands.; ^9^ Electron Microscopy Center Amsterdam, Amsterdam UMC location University of Amsterdam, Amsterdam, Netherlands.; ^10^ Complex Carbohydrate Research Center, University of Georgia, Athens, GA, USA.; ^11^ Department of Global Health, Amsterdam Institute for Global Health and Development, Amsterdam UMC, University of Amsterdam, Amsterdam, Netherlands.; ^12^ Netherlands Reference Laboratory for Bacterial Meningitis (NRLBM), Amsterdam UMC location AMC, Amsterdam, Netherlands.

## Abstract

*Streptococcus suis* is a largely neglected but emerging bacterial zoonotic pathogen of global concern for animal welfare, antibiotic resistance development, and human health. No effective vaccines are now available. Here, we identified and characterized the function and structure of two cell wall polysaccharide variants in pathogenic *S. suis* strains using genetic deletion and (heterologous) complementation, lectin staining, glycan composition analysis, and specialized NMR spectroscopy. Both glycan variants were anionic polymers that differed in the presence of glucose in the side chain as a result of allelic variation in a glycosyltransferase gene. Deletion of this variable glycosyltransferase revealed an identical glycan “core” and affected *S. suis* morphology and lysozyme resistance. Immunization of pigs with this core domain elicited antibodies that recognized antigenically diverse pathogenic *S. suis* strains and induced complement deposition on encapsulated pathogenic *S. suis* strains. This study provides valuable insights for developing next-generation glycoconjugate vaccines, whereby a single-glycan target could protect against the emerging zoonotic pathogen *S. suis*.

## Teaser

Identification of a conserved and immunogenic glycan core as a vaccine target for the zoonotic pathogen *Streptococcus suis*.

## INTRODUCTION

*Streptococcus suis* is a ubiquitous commensal of the porcine upper respiratory tract that can cause severe infections such as meningitis, septicemia, and arthritis in pigs resulting in high mortality and substantial economic losses (*1*, *2*). In addition to infections in livestock, zoonotic *S. suis* infections are increasingly reported globally and have been identified as the main cause of adult bacterial meningitis in several South-East Asian countries (*3*–*5*). *S. suis* infections are a key driver of antibiotic usage in pigs, thereby contributing to the emergence of multidrug-resistant *S. suis* strains and development of antibiotic resistance in other bacteria (*6*, *7*). Overall, there is urgent need for innovative preventive measures, particularly vaccines, to prevent *S. suis* infections and improve animal welfare, protect human health, and reduce antibiotic resistance development (*5*, *8*).

Bacterial surface glycans serve as a protective layer around the bacterium, forming an important physical interface between host and pathogen. Capsular polysaccharides (CPS) form the outermost layer and are important virulence factors, enabling evasion of immune-mediated killing by complement and neutrophils. CPS-based glycoconjugate vaccines induce high functional immunoglobulin G (IgG) antibody titers and have been instrumental in reducing mortality and morbidity caused by *Streptococcus pneumoniae*, *Haemophilus influenzae*, and *Neisseria meningitidis* (*9*, *10*), especially in young children. However, bacterial pathogens can express a range of structurally diverse CPS (e.g., *S. pneumoniae*), resulting in antigenic variation. Limited cross-serotype protection has offset vaccine-induced reductions in disease incidence as vaccine-targeted serotypes are replaced by other nonvaccine covered serotypes, which can also cause disease (*11*, *12*). In line with human glycoconjugate vaccines, a CPS-conjugate vaccine has been successful in preventing *S. suis* infection in pigs in preclinical studies (*13*). Similar to human CPS-conjugate vaccines, this vaccine only provided serotype-specific protection, covering only one of the 29 *S. suis* CPS serotypes (*1*). The application of CPS-based vaccines against *S. suis* is further complicated by frequent capsule switching events in the *S. suis* population (*14*), facilitating strain replacement under selective immunological pressure. A strategy is required to overcome the serotype limitations of traditional CPS-glycoconjugate vaccines, ensuring broad-spectrum protection against *S. suis* infections, support healthy farming, lower antibiotic use in livestock, and indirectly reduce zoonotic infections in humans (*14*).

Alongside CPS, Gram-positive bacteria express cell wall–anchored glycan polymers on their surface at high density, which often exhibit less structural variation compared to CPS. Well-studied glycopolymer families include wall teichoic acids in staphylococci and rhamnose-rich polysaccharides (RPS) in streptococcal species (*15*, *16*). Historically, structural variation in RPS composition was used to classify hemolytic streptococci into Lancefield groups (e.g., Lancefield groups A, B, C, and G) for diagnosing infections (*15*, *17*) and to differentiate *Streptococcus mutans* into distinct serotypes (i.e., serotypes c, e, f, and k) (*18*, *19*). However, the role of RPS extends well beyond that of a structural cell wall component and diagnostic agent, fulfilling critical functions in streptococcal cell division and bacterial virulence (*20*–*24*). The conserved nature of the RPS in *Streptococcus pyogenes* (i.e., group A *Streptococcus*) has led to incorporation of native or modified RPS molecules into multicomponent vaccines now in clinical development (*22*, *25*).

Similar to *S. pyogenes* and *S. mutans*, we hypothesized that *S. suis* expresses RPS molecules with limited structural variation as compared to CPS variation. Here, we characterized the biosynthesis gene cluster, glycosyl linkage patterns, and function of RPS in the zoonotic pathogen *S. suis*. We identified two structural RPS variants in pathogenic *S. suis* lineages, which share a conserved glycan motif and elicited antibodies that recognized a wide range of pathogenic *S. suis* strains in a serotype-independent manner. Moreover, the antibodies were functional as evidenced by inducing antibody-mediated complement deposition on encapsulated *S. suis* strains, thereby also providing evidence that RPS is accessible in the presence of capsule. These findings pave the way for the development of next-generation glycoconjugate vaccines, whereby a single-glycan target could protect against the genetically and antigenically diverse zoonotic pathogen *S. suis*.

## RESULTS

### Identification of *S. suis* dTDP-rhamnose and RPS biosynthesis gene clusters

**Fig. 1.**
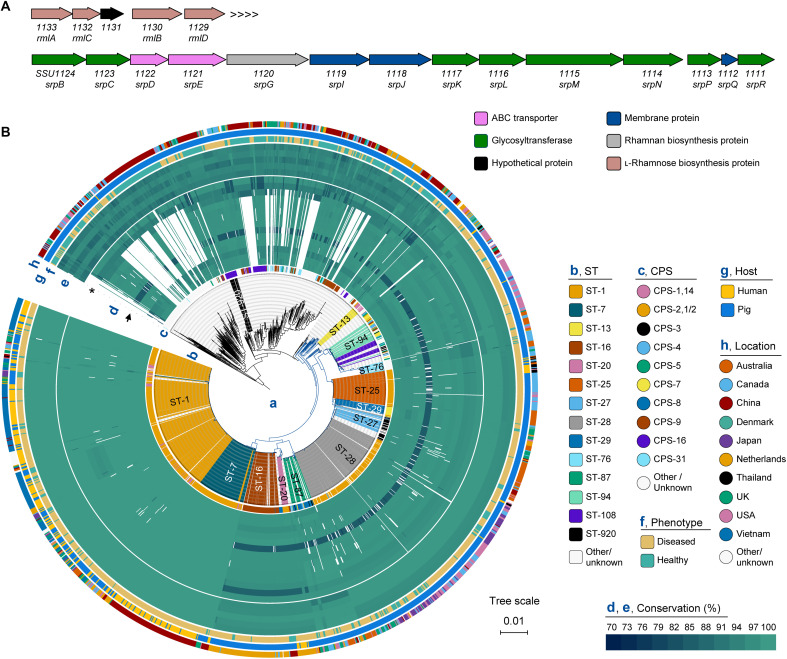
Identification and overview of the RPS biosynthetic gene cluster in the *S. suis* population. (**A**) Schematic representation of the *S. suis* dTDP-rhamnose gene cluster (top) and RPS gene cluster (bottom) in strain P1/7 genome (National Center for Biotechnology Information accession: NC_012925.1). The arrows are scaled according to gene size, with each predicted function represented by a different color. (**B**) Analysis of the putative dTDP-rhamnose and RPS biosynthesis gene cluster in a collection of 1719 *S. suis* genomes. (a) Core genome phylogenetic tree. Branches of the pathogenic lineages are colored blue. (b) Sequence type (ST); (c) serotype-based on capsular polysaccharide (CPS) composition. (d and e) Conservation of RPS (d) and rhamnose (e) biosynthesis genes. The dTDP-rhamnose and RPS biosynthesis genes of P1/7 were compared to gene homologs in all other genomes using BLAST. For hits with >90% coverage and >80% identity, conservation was calculated by multiplying coverage and identity and visualized using heatmaps. From outer to inner ring of the RPS cluster (d) is *srpBCDEGIJKLMNPQR*, respectively. Asterisk, *srpC*; arrow, *srpL*. From the outer to inner ring of the dTDP-rhamnose biosynthesis cluster (e) is *rmlA*, *rmlC*, *SSU1131*, *rmlB*, and *rmlD*, respectively. (f) Disease phenotype of the host. (g) Host species; (h) country where strain was isolated (location).

The genetic loci encoding the genes required for the biosynthesis of *S. pyogenes* RPS [also known as group A carbohydrate (GAC)] and *S. mutans* serotype c RPS [also known as serotype c carbohydrate (SCC)] have been identified and characterized previously (fig. S1A) (*20*, *22*). The putative *S. suis* RPS biosynthesis orthologs *SSU1124*-*1111* form a 14-gene cluster (Fig. 1A), herein designated *srpBCDEGIJKLMNPQR.* This cluster showed a low guanine-cytosine content (fig. S1B), suggesting acquisition by horizontal gene transfer. The genes encode glycosyltransferases (including a rhamnan biosynthesis protein), an ABC transporter, and membrane proteins (Fig. 1A and table S1). In contrast to the GAC and SCC genetic loci (*20*, *22*), the *S. suis* RPS gene cluster lacked genes required for dTDP-rhamnose biosynthesis (fig. S1A). However, a five-gene cluster, which includes putative orthologs of previously identified and characterized dTDP-rhamnose biosynthesis genes *rmlA*-*D* and a gene of unknown function, was identified upstream of *srpBCDEGIJKLMNPQR* in the *S. suis* P1/7 genome (Fig. 1A). We also identified a gene, termed *srpO* (*SSU1672*; table S1), as an ortholog of *S. pyogenes gacO*, *Streptococcus agalactiae gbcO*, and *S. mutans rgpG*, which encode a UDP-*N*-acetylglucosamine (UDP-GlcNAc): undecaprenol-phosphate GlcNAc-phosphate (GlcNAc-P-P-Und) transferase that catalyzes the first and essential step of GAC, GBC (group B carbohydrate), and SCC biosynthesis, respectively (*21*–*23*). Similar to other streptococcal species, *srpO* is located separately from the putative RPS gene cluster.

### Glycosyltransferases SrpC and SrpL show allelic diversity in pathogenic *S. suis* lineages

The level of RPS structural variation differs between species. For example, the GAC biosynthesis gene cluster is highly conserved among the *S. pyogenes* population (*22*, *26*), whereas the *S. mutans* RPS gene cluster has four identified variants corresponding to its four serotypes (*19*, *27*). To investigate the conservation of the RPS genes across the genetically diverse *S. suis* population, we examined the five dTDP-rhamnose biosynthesis genes and the 14 genes of the putative RPS gene cluster by ABRicate in a collection of 1719 publicly available *S. suis* genomes (*28*). The dTDP-rhamnose biosynthesis genes were ubiquitously present in all genomes and showed little sequence variation (Fig. 1B). In contrast, the composition of the RPS gene cluster varied within the *S. suis* population (Fig. 1B). In the pathogenic *S. suis* lineages, the putative RPS biosynthesis gene cluster was conserved with regard to the number and organization of RPS genes but showed allelic diversity in the two putative glycosyltransferase-encoding genes, *srpC* and *srpL* (Fig. 1B and fig. S2A). To quantitatively assess gene conservation, we defined a conservation index as the product of gene identity and coverage. In the highly pathogenic and zoonotic ST-1 and ST-7 lineages, all RPS genes showed >91.5% conservation to those in reference strain P1/7. By contrast, the Dutch invasive ST-16 and ST-20 lineages showed <86.5% conservation in two glycosyltransferase-encoding genes *srpC* and *srpL* compared to P1/7. Sequence variation in *srpL* was also observed in strains belonging to the ST-25 and ST-28 lineages, which are dominant sequence types (STs) in Australia and North America (Fig. 1B). These findings suggest that the RPS glycan composition or structure may differ between pathogenic *S. suis* lineages. RPS genes in nonpathogenic lineages (*14*) had limited homology to those in P1/7 (Fig. 1B and fig. S2B), suggesting structural differences in RPS expressed by pathogenic and nonpathogenic *S. suis* lineages.

**Fig. 2.**
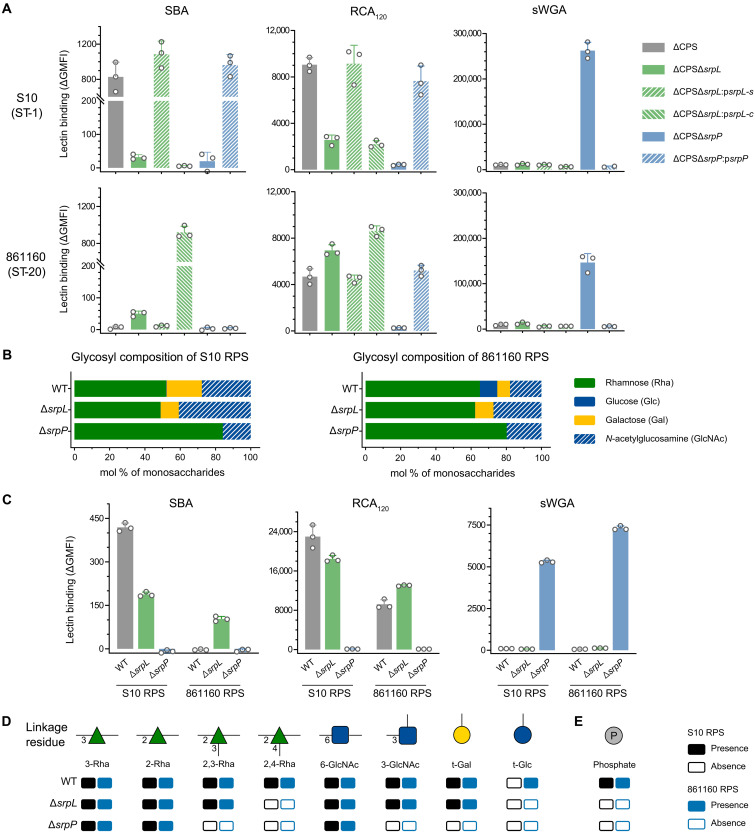
Lectin staining and glycan composition analysis of *S. suis* RPS. (**A**) Binding of plant lectins to surface structures of capsule-deficient *S. suis* strains (ΔCPS). SBA binds to *N*-acetylgalactosamine (GalNAc) and, to a lesser extent, galactose (Gal); RCA_120_ binds to both Gal and GalNAc; sWGA has a special affinity to *N*-acetylglucosamine (GlcNAc). Data from biological triplicates are presented as mean values ± SD. (**B**) Glycosyl composition analysis by GC-MS of TMS (trimethylsilyl) derivatives of methyl glycosides of *S. suis* RPS from S10 and 861160 released by mild acid hydrolysis after chemical *N*-acetylation. (**C**) Plant lectin binding to isolated RPS from *S. suis* S10 and 861160. Data show technical triplicates (mean values ± SD) and are representative for two independent experiments. (**D** and **E**) Presence and absence of the most abundant glycosyl linkage residues (D) and phosphate (E) of *S. suis* RPS from S10 and 861160. Glycosyl linkage residues were analyzed by GC-MS of partially methylated alditol acetate derivatives. Phosphate was determined by malachite green assay following hydrolysis with hydrochloric acid and digestion with alkaline phosphatase. Original data is in tables S2 and S3.

To investigate whether *S. suis* strains from different pathogenic lineages express structurally different RPS, we assessed the binding of a panel of plant lectins to ST-1 strain S10 and ST-20 strain 861160. Capsule-deficient mutants were used to exclude interference from lectin binding to CPS. Soybean agglutinin (SBA) and *Ricinus communis* agglutinin I (RCA I or RCA_120_), which bind to *N*-acetylgalactosamine (GalNAc) and galactose (Gal) with different affinities, demonstrated binding to S10 ΔCPS (Fig. 2A). In contrast, 861160 ΔCPS was only stained by RCA_120_ (Fig. 2A), suggesting that these two strains differ in their RPS composition. To investigate whether structural diversity was linked to the observed allelic diversity in *srpC* and *srpL*, we attempted to delete *srpC* and *srpL* in both strains by homologous recombination. Despite multiple attempts, *srpC* deletion was unsuccessful, suggesting that *srpC* is essential for *S. suis* viability. Deletion of *srpL* in S10 reduced the binding to both SBA and RCA_120_, whereas *srpL* deletion in 861160 increased binding to SBA and RCA_120_ (Fig. 2A). Complementary, plasmid complementation of ΔCPSΔ*srpL* mutants with the homologous *srpL* gene (p*srpL-s*) restored the binding to levels observed in the parent strains, whereas cross-complementation (p*srpL-c*) did not (Fig. 2A). These results suggest that RPS has different compositions in strains S10 and 861160, potentially resulting from genetic differences in *srpL*.

We also deleted *srpP*, a homolog of *S. pyogenes gacI*, which encodes a *N*-acetylglucosamine-phosphate-undecaprenol (GlcNAc-P-Und) synthase and is essential for biosynthesis of the immunodominant GlcNAc side chain in *S. pyogenes* GAC (*22*, *29*). Deletion of *srpP* resulted in loss of both RCA_120_ and SBA binding but conferred binding of succinylated wheat germ agglutinin (sWGA), which has affinity to GlcNAc (Fig. 2A). These observations indicate that *S. suis* RPS contains Gal or GalNAc as terminal moieties and GlcNAc as intermediate moiety. Last, we attempted to generate a complete RPS-deficient mutant by inactivating *srpO*, but similar to other streptococci, we were unable to obtain mutants after multiple attempts, suggesting that *srpO* is essential to *S. suis*.

### *S. suis* RPS has two structural variants but contains a conserved glycan motif

To gain more detailed insight into the structural composition of *S. suis* RPS, we released RPS from the cell wall of S10ΔCPS mutant by mild acid hydrolysis after chemical *N*-acetylation, followed by purification by size exclusion chromatography (*30*). The obtained fraction contained rhamnose, hexose, and phosphate (fig. S3A). Further purification of isolated RPS by diethylaminoethyl (DEAE) ion-exchange chromatography showed the presence of DEAE-bound and DEAE-unbound fractions (fig. S3B), indicative of a charged and uncharged fraction, respectively. Gas chromatography–mass spectrometry (GC-MS)–based glycosyl composition analysis revealed that the two fractions of the S10 wild-type (WT) RPS contained the same monosaccharides, i.e., Rha, Gal, and GlcNAc at a molar ratio of 5.2:2.0:2.7 (Fig. 2B and fig. S3C). By contrast, the 861160 WT RPS, prepared in a similar manner, contained Rha, Glc, Gal, and GlcNAc in a molar ratio of 6.5:1.0:0.7:1.8 (Fig. 2B). These results confirm that the S10 WT RPS and the 861160 WT RPS differ in their glycan composition by the absence or presence of Glc, respectively.

Since deletion of *srpL* and *srpP* changed plant lectin binding (Fig. 2A), we performed GC-MS–based glycosyl composition analysis on the RPS of these mutant strains. RPS isolated from ΔCPSΔ*srpL* mutants showed decreased Gal content in S10 and complete absence of Glc but no difference in Gal in 861160 (Fig. 2B). The isolated RPS molecules were also probed by plant lectins (Fig. 2C), and the results were consistent with lectin binding to whole bacteria and glycosyl composition analysis. RPS isolated from the S10 Δ*srpP* and 861160 Δ*srpP* deletion strains revealed an identical glycan composition, consisting of Rha and GlcNAc and lacking Gal and Glc (Fig. 2B). Correspondingly, the samples showed identical plant lectin staining profiles (Fig. 2C). Overall, these observations confirm that the RPS glycan composition is different between strain S10 and 861160 and can be attributed to genetic variation in *srpL*.

To investigate the structural differences in RPS from WT and related deletion strains in S10 and 861160, a detailed glycosyl linkage analysis of RPS from S10 and 861160, using GC-MS of partially methylated alditol acetate derivatives, was performed. Both the S10 and 861160 WT RPS contained 3-Rha, 2-Rha, 2,3-Rha, 2,4-Rha, 6-GlcNAc, 3-GlcNAc, and terminal Gal as the most abundant linkage residues, whereas the 861160 WT RPS contained additional terminal Glc (Fig. 2D). Deletion of *srpL* resulted in the loss of 2,4-Rha in both strains and loss of terminal Glc in 861160 (Fig. 2D), in line with results from the composition analysis (Fig. 2B). Upon deletion of *srpP*, RPS showed the presence of 3-Rha, 2-Rha, and 6-GlcNAc but loss of 2,3-Rha, 2,4-Rha, 3-GlcNAc, and terminal Gal in both strains and the absence of terminal Glc in 861160 (Fig. 2D). Furthermore, nuclear magnetic resonance (NMR) spectral data from ^1^H,^13^C–heteronuclear single-quantum coherence (HSQC) experiments indicate that Δ*srpP* RPS from both S10 and 861160 contained similar linear polysaccharides consisting of trisaccharide repeating units. Additional analysis of a *F*_2_-coupled ^1^H,^13^C-HSQC spectrum of Δ*srpP* RPS showed that the one-bond coupling constants of the anomeric atoms were^1^*J*_C1,H1_ = 171 to 177 Hz. Therefore, we concluded that the three sugar residues have the α-anomeric configuration. The six-substituted GlcNAc residue (Fig. 2D) showed two cross-peaks at δ_C6_/δ_H6a_ 67.22/3.74 and δ_C6_/δ_H6b_ 67.22/3.97 in the multiplicity-edited ^1^H,^13^C-HSQC spectrum. These ^1^H NMR resonances of the hydroxymethyl group were used as the starting point for further elucidating the structure of Δ*srpP* RPS based on a ^1^H,^1^H–nuclear Overhauser effect spectroscopy NMR experiment (*31*). The anomeric proton at δ_H1_ 4.91 showed, inter alia, transglycosidic nuclear Overhauser effect (NOE) cross-peaks to δ_H6_ 3.74 and 3.97 as well as an intraresidue NOE to δ_H2_ 4.01 (fig. S4), consistent with the structural element α-L-Rha*p*-(1 → 6)-α-D-Glc*p*NAc. The anomeric proton at δ_H1_ 5.01 showed an interresidue NOE to δ_H2_ 4.01 and an intraresidue NOE to δ_H2_ 4.07, revealing the structural element α-L-Rha*p*-(1 → 2)-α-L-Rha*p*. With this structural information, we interpreted the additional NOE observed from the anomeric proton at δ_H1_ 4.91, viz., to δ_H5_ 3.78 of the subsequent residue in the α-(1 → 2)–linked rhamnose disaccharide entity, as observed previously in a disaccharide (*32*) as well as in the RPS of *Streptococcus uberis* (*33*). The anomeric proton at δ_H1_ 5.02 showed an interresidue NOE to δ_H2_ 4.07 and an intraresidue NOE to δ_H2_ 3.94, revealing the structural element α-D-Glc*p*NAc-(1 → 2)-α-L-Rha*p*. Together, these results defined the repeating unit of the Δ*srpP* RPS backbone as →2)-α-L-Rha*p*-(1 → 2)-α-L-Rha*p*-(1 → 6)-α-D-Glc*p*NAc-(1→. The presence of a derivative arising from three-linked rhamnose residues in the Δ*srpP* RPS material (Fig. 2D) is presumed to originate from an adaptor region in the RPS (*34*). RPS of Δ*srpL* mutants additionally contained similar branched side chains resulting in hexasaccharide repeating units. Thus, RPS of Δ*srpL* mutants represents a conserved glycan motif, which may serve as a good candidate for a broad-spectrum vaccine against *S. suis*.

### Glycerol phosphate is linked to different sugars in ST-1 strain S10 and ST-20 strain 861160

**Fig. 3.**
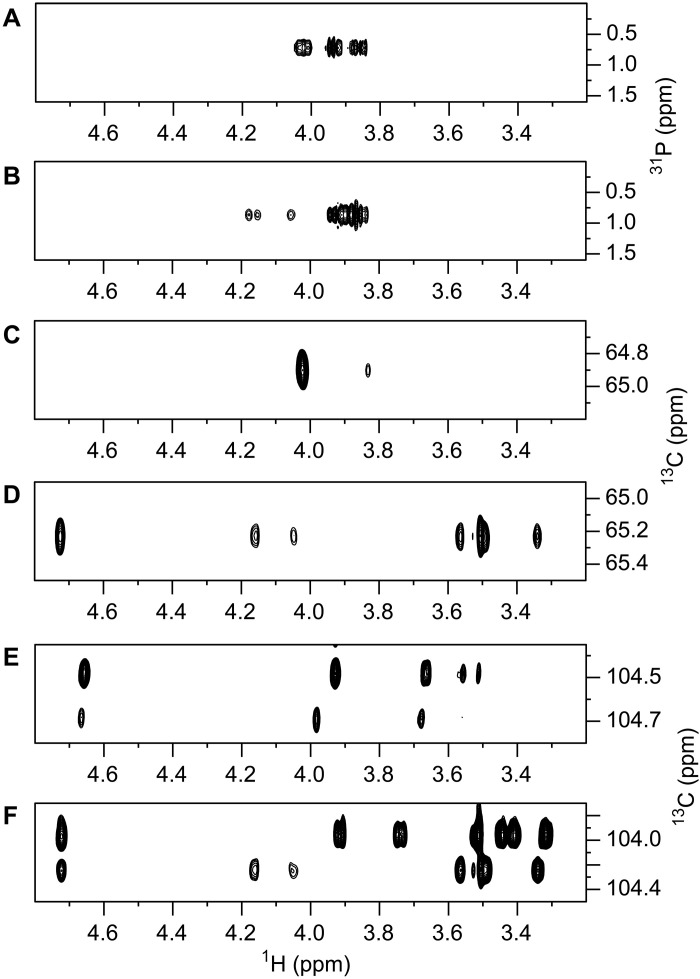
Selected NMR spectral regions of RPS from S10 and 861160. (**A** and **B**) ^1^H,^31^P-HMBC NMR spectrum of RPS from S10 (A) and RPS from 861160 (B), identifying phosphodiester-linked entities. Spectral region from a ^1^H,^13^C-HSQC-TOCSY NMR experiment (mixing time of 200 ms) showing correlations from C6 of a hexopyranose residue substituted by *sn*-Gro-1-*P* at the hydroxymethyl group for (**C**) RPS from S10 revealing only a limited number of cross-peaks consistent with a *galacto*-configuration of the sugar and (**D**) RPS from 861160 displaying cross-peaks for a full ^1^H,^1^H spin system for a hexose, including the anomeric proton, consistent with a *gluco*-configuration of the sugar. (**E**) Spectral region from the ^1^H,^13^C-HSQC-TOCSY NMR experiment for the RPS from S10 showing correlations from anomeric carbons of hexopyranose residues devoid of a GroP substituent (major, δ_H1_/δ_C1_ 4.66/104.47, ^1^*J*_C1,H1_ 162 Hz) and carrying a GroP substituent (minor, δ_H1_/δ_C1_ 4.67/104.67, ^1^*J*_C1,H1_ 162 Hz) revealing only a limited number of cross-peaks consistent with a *galacto*-configuration of the sugar and (**F**) the corresponding spectral region from the ^1^H,^13^C-HSQC-TOCSY NMR experiment for the RPS from 861160 displaying cross-peaks for full ^1^H,^1^H spin systems, including hydroxymethyl protons, of hexopyranose residues devoid of a GroP substituent (major, δ_H1_/δ_C1_ 4.723/103.96, ^1^*J*_C1,H1_ 165 Hz) and carrying a GroP substituent (minor, δ_H1_/δ_C1_ 4.725/104.24, ^1^*J*_C1,H1_ 164 Hz) consistent with a *gluco*-configuration of the sugar.

GAC and SCC carry a negative charge because of a glycerol phosphate (GroP) modification, which increases susceptibility to cationic antimicrobial peptides and cationic antibacterial proteins like human group IIA–secreted phospholipase A_2_ (hGIIA) (*24*, *26*, *30*). Similarly, both *S. suis* WT RPS variants were negatively charged and contained phosphate (Fig. 2E and fig. S3), suggesting that *S. suis* RPS may similarly be modified with GroP. However, phosphate was not detected in Δ*srpL* or the Δ*srpP* RPS from both S10 and 861160 (Fig. 2E), implying that the terminal Gal transferred by SrpL in S10 or terminal Glc transferred by SrpL in 861160 served as the GroP acceptor. To confirm the presence and location of GroP, purified native RPS from both strains were analyzed by NMR spectroscopy. The S10 RPS contained a phosphodiester group, as indicated by the NMR chemical shift (*35*), δ_P_ 0.71, and a ^1^H,^31^P–heteronuclear multiple-bond correlation (HMBC) experiment revealed correlations to protons in the spectral region 4.05 to 3.80 parts per million (ppm; Fig. 3A). For 861160 RPS, the corresponding NMR data were δ_P_ 0.86 and 4.20 to 3.80 ppm (Fig. 3B). Furthermore, in the ^13^C NMR spectra, resonances with closely similar chemical shifts were observed, viz., δ_C_ 67.20 (*J*_CP_ 5.6 Hz), δ_C_ 71.55 (*J*_CP_ 7.6 Hz), and δ_C_ 63.02 for the S10 RPS and δ_C_ 67.17 (*J*_CP_ 5.6 Hz), δ_C_ 71.56 (*J*_CP_ 7.6 Hz), and δ_C_ 63.05 for the 861160 RPS. These NMR findings resemble those from *S. pyogenes* GAC, in which the substituent *sn*-Gro-1-*P* is linked to position 6 of a β-D-Glc*p*NAc residue as a side chain in the polysaccharide (*24*). Similarly, in *S. mutans* SCC, *sn*-Gro-1-*P* is also linked to position 6, although, in this case, to the α-D-Glc*p* (*27*).

Additional NMR analysis confirmed that both RPS variants contained terminal Gal residues, whereas only the 861160 RPS contained terminal glucose residue(s) (vide infra). The ^1^H,^13^C-HSQC NMR spectra of the two RPS structures showed cross-peaks of hydroxymethyl groups shifted to higher ^13^C NMR chemical shifts, indicative of phosphorylation (*36*), with an increase of ~+3 ppm to ~65 ppm. The ^1^H,^13^C-HSQC-TOCSY (total correlation spectroscopy) NMR spectrum of the S10 RPS showed a cross-peak at δ_H5_ 3.83 (Fig. 3C) besides the cross-peak at δ_H6_/δ_C6_ 4.02/64.90, consistent with a Gal residue having δ_C5_ 74.41. The corresponding ^1^H,^13^C-HSQC-TOCSY NMR spectrum of 861160 RPS showed cross-peaks at δ_H5_ 3.57 and δ_H1_ 4.725 (Fig. 3D) besides cross-peaks at δ_H6a_/δ_C6_ 4.05/65.23 and δ_H6b_/δ_C6_ 4.16/65.23, consistent with a Glc residue having δ_C5_ 75.58 (*J*_C5,P_ 8.3 Hz). The assignments of Gal and Glc residues in S10 and 861160 RPS, respectively, were consistent with the lower number of cross-peaks in S10 (Fig. 3E) compared to the full ^1^H,^1^H spin system of the hexose in 861160 RPS (Fig. 3F). Further analysis confirmed that S10 RPS contained a Gal residue substituted at O6 by an *sn*-Gro-1-*P* group. This was supported by the presence of a cross-peak, δ_H5_/δ_C4_ 3.83/69.02, in a ^1^H,^13^C-H2BC NMR spectrum, indicating a large ^2^*J*_H5,C4_ coupling constant, as observed in Gal and lactose (*37*). The absolute configuration of the substituent was assumed to be *sn*-Gro-1-*P* similar to that found in *S. pyogenes* GAC (*24*). Thus, complementary NMR analysis showed that the RPS of S10 and 861160 carry the GroP substituent at O6 of Gal and Glc residues, respectively, both of which are β-linked, as inferred from the magnitude of their ^1^*J*_C1,H1_ coupling constant (NMR data given in the legend to Fig. 3). The identified *S. suis* RPS biosynthesis gene cluster lacked an apparent ortholog of GroP transferases encoding *gacH* and *sccH*, which are essential for the transfer of GroP on GAC and SCC, respectively (Fig. 1A and fig. S1A) (*20*, *24*). Therefore, the GroP transferase responsible for the GroP modification of *S. suis* RPS is likely located separately from the RPS biosynthesis gene cluster and remains to be identified.

### RPS side chain is important for *S. suis* biology

**Fig. 4.**
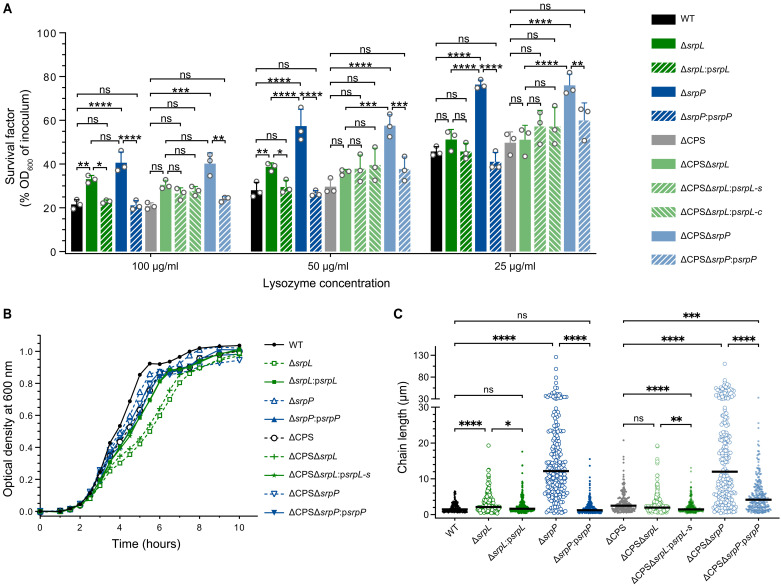
RPS side chain is important for *S. suis* lysozyme susceptibility and cell division. (**A**) Survival of different *S. suis* S10 WT, capsule-deficient mutant, and isogenic *srpL* and *srpP* deletion mutants and complementation strains in the presence of different concentrations of lysozyme. ΔCPS represents a capsule-deficient background. Data from biological triplicates are presented as mean values ± SD. *P* values were calculated by two-way analysis of variance (ANOVA) with Tukey’s multiple comparisons test. (**B**) Growth curves of S10 WT, capsule-deficient mutant, and isogenic *srpL* and *srpP* deletion mutants and complementation strains. Data of Δ*srpL* and Δ*srpP* mutants were collected from three and two independent experiments, respectively. Representative curves are shown. (**C**) Quantification of chain length (in micrometers) of S10 WT and isogenic *srpL* and *srpP* deletion mutants and complementation strains. Overnight culture in THY with gentle shaking was used for Gram staining (Fig. 5, left column), and multiple pictures of every single strain were taken under 40× microscope objective. The quantification of chain length was performed by measuring all bacterial chains in every single picture using ImageJ, until at least 200 chains were measured. The line represents the median value. *P* values were calculated by Kruskal-Wallis test with Dunn’s multiple comparisons test. *P ≤ 0.05, **P ≤ 0.01, ***P ≤ 0.001, and ****P ≤ 0.0001. ns, not significant.

In *S. pyogenes*, the GAC-GlcNAc side chain and the GroP modification affect the susceptibility of *S. pyogenes* to human antimicrobial defenses, such as antimicrobial peptides, hGIIA, and lysozyme (*22*, *30*, *38*). To determine whether specific RPS epitopes affect *S. suis* immune resistance, we assessed lysozyme susceptibility. Both the Δ*srpL* and Δ*srpP* mutants showed increased resistance to lysozyme compared to the isogenic WT strain (Fig. 4A), with mutants lacking the entire RPS side chain (Δ*srpP* and ΔCPSΔ*srpP*) being most resistant (Fig. 4A). These results indicate that the RPS side chain contributes to lysozyme susceptibility in *S. suis*, in line with observations in other streptococci.

**Fig. 5.**
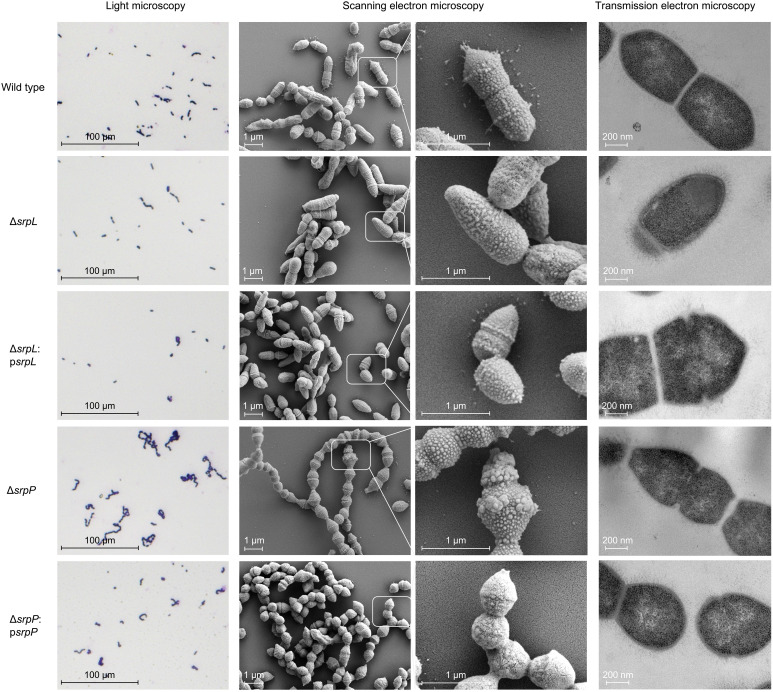
RPS side chain is important for *S. suis* morphology. Representative microscopy images of encapsulated S10 WT and isogenic *srpL* and *srpP* deletion mutants and complementation strains. Light microscopy (left column) images were taken on Gram-stained stationary phase bacteria from overnight culture in THY; scanning (middle two columns) and transmission (right column) electron microscopy images were taken on exponential phase bacteria (OD ≈ 0.4 in THY).

To further explore the function of the RPS side chain in *S. suis* biology, we compared the growth and morphology of S10 Δ*srpL* and Δ*srpP* mutants to S10 WT. The S10 Δ*srpL* mutant, which lacks the Gal-GroP side chain, showed slower growth in rich culture media (Fig. 4B) and increased chain length (Fig. 4C). By contrast, the Δ*srpP* mutants, which only expressed the RPS trisaccharide backbone, displayed normal growth but significantly increased chain length (Figs. 4C and 5) and enhanced self-aggregation after overnight culture (fig. S5). Scanning electron microscopy showed that the encapsulated Δ*srpP* mutant is cone shaped, with some swollen, small blebs, and fractional cells (Fig. 5, middle column, and fig. S6, A and B), but transmission electron microscopy (TEM) showed that septa were being formed (Fig. 5, right column). In contrast, all ΔCPS mutants showed a smoother surface, and the ΔCPSΔ*srpL* and ΔCPSΔ*srpP* mutants showed longer cell length compared to the ΔCPS parent strain (fig. S6C, right column). Plasmid-based complementation of deleted genes restored growth, chain length, and cell length of the mutants but not the cell shape of the encapsulated Δ*srpP* mutant (Figs. 4, B and C, and 5, and fig. S6C). Together, these observations indicate that the RPS side chain is important for *S. suis* morphology, chaining, and growth.

### The core RPS motif induces functional IgG antibodies in piglets that are cross-reactive with diverse pathogenic and encapsulated *S. suis* isolates

**Fig. 6.**
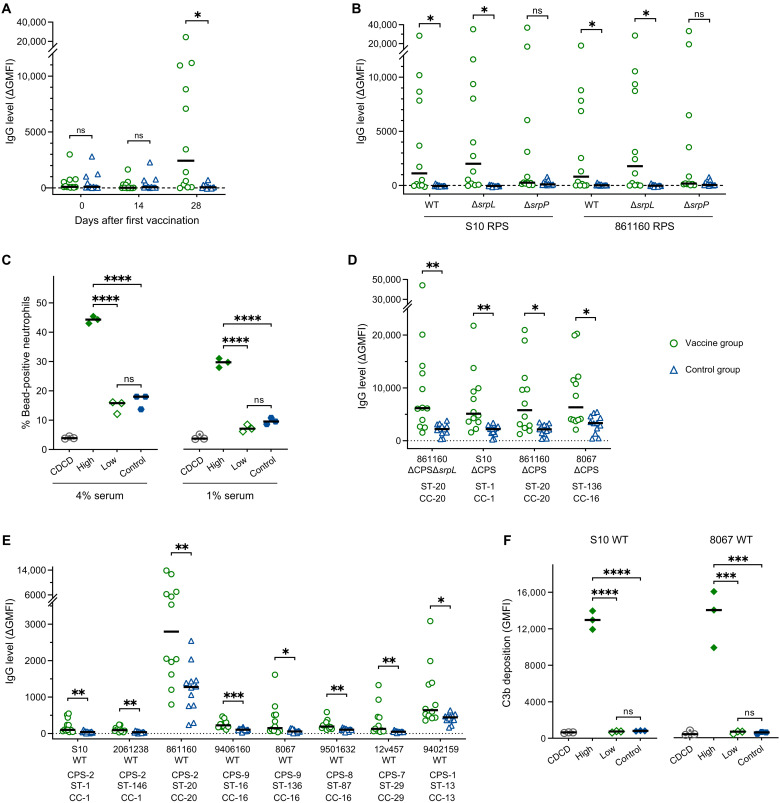
The conserved RPS motif is immunogenic and induces functional antibodies with broad reactivity against *S. suis*. (**A**) IgG responses against a conserved RPS motif isolated from 861160 ΔCPSΔ*srpL* in piglets after two vaccinations when administrated as a glycoconjugate vaccine. Three-week-old piglets (*n* = 12 per group) were immunized twice with a 2-week interval using adjuvanted RPS-glycoconjugate. IgG responses to the immunizing glycan were assessed in serum samples collected on days 0, 14, and 28. (**B**) Reactivity of vaccine-induced IgG against different RPS variants, including the WT structures, using sera from day 28. (**C**) Phagocytosis of 861160 WT RPS-coated beads by porcine neutrophils in the presence of pooled serum from the highest (high) and lowest (low) three responders in the vaccine group and a serum pool from all control animals (control). Heat-inactivated pooled serum from CDCD piglets was used as a negative control. Data represent technical triplicates and are representative for two donors. (**D** and **E**) Reactivity of RPS-induced IgG on day 28 against CPS-deficient (D) and encapsulated WT *S. suis* strains (E) representing different sequence and serotypes. (**F**) Complement deposition, measured as C3b deposition, on encapsulated *S. suis* strains (S10 and 8067) in the presence of 10% CDCD serum as an active complement source. Serum samples (4%) were the same as (C). Data show technical triplicates and are representative for two independent experiments. For [(A), (B), (D), and (E)], sera were used at a final concentration of 1%. *P* values were calculated by Mann-Whitney test with Holm-Šídák’s multiple comparisons test. CPS, capsular polysaccharide (serotype); CC, clonal complex. For [(C) and (F)], *P* values were calculated by one-way ANOVA with Tukey’s multiple comparisons test. Lines represent the median values. *P ≤ 0.05, **P ≤ 0.01, ***P ≤ 0.001, and ****P ≤ 0.0001.

Thus far, structural analysis of RPS from two pathogenic *S. suis* strains revealed limited structural variation and the presence of a shared glycan core motif consisting of Rha, GlcNAc, and Gal. We hypothesized that this core glycan domain could elicit broadly reactive antibodies that would be unaffected by subtle glycan variations in natively expressed RPS. To test this, RPS was purified from the 861160 ΔCPSΔ*srpL* mutant and conjugated to the carrier protein CRM_197_ using click chemistry [copper(I)–catalyzed azide-alkyne cycloaddition (CuAAC)] (*39*) to generate a glycoconjugate construct (fig. S7A). The glycoconjugate was adjuvanted with X-Solve, and 3-week-old piglets were immunized intramuscularly twice with a 2-week interval. No increased antibody reactivity to either the carrier protein or RPS was observed on day 14 after the first immunization (Fig. 6A). However, on day 28, 2 weeks after the second immunization, 7 (58.3%) out of 12 piglets showed IgG responses against the immunizing glycan (Fig. 6A), while all piglets developed IgG against CRM_197_ (fig. S7B). In contrast, no IgG reactivity against the carrier protein or the RPS glycan was detected in the control group (Fig. 6A and fig. S7B). IgG antibodies elicited by the RPS-conjugate were reactive against purified WT, Δ*srpL*, and Δ*srpP* RPS of both S10 and 861160 (Fig. 6B). In addition, pooled serum from high IgG responders (*n* = 3) significantly enhanced antibody-mediated phagocytosis of 861160 WT RPS-coated beads by porcine neutrophils compared to pooled serum from low IgG responders (*n* = 3), unvaccinated controls (*n* = 12), and cesarean-derived, colostrum-deprived (CDCD) piglets (*n* = 6; Fig. 6C), which is antibody deficient. Addition of a complement source resulted in high unspecific C3b deposition independent of RPS-reactive antibodies (fig. S8A). These data show that high levels of RPS-specific antibodies are functional as opsonins for phagocytosis by pig neutrophils.

To investigate whether the elicited RPS-reactive antibodies would also be able to recognize natively expressed RPS on the surface of *S. suis*, piglet sera were incubated with representative *S. suis* strains from various genetic backgrounds within pathogenic lineages. The RPS-reactive IgG bound to four different CPS-deficient *S. suis* strains (Fig. 6D), as well as eight encapsulated WT strains representing five disease-associated serotypes and eight STs (Fig. 6E). Although capsule expression reduced RPS antibody binding to *S. suis*, the pooled serum from high IgG responders significantly promoted C3b deposition on the surface of encapsulated *S. suis* strains compared to pooled serum from low IgG responders and unvaccinated controls or pooled CDCD serum (Fig. 6F). In the absence of capsule, all tested serum pools, including CDCD serum lacking *S. suis*–specific antibodies, resulted in high unspecific C3b deposition (fig. S8B). Overall, these results show that antibodies induced by the RPS core domain are able to access RPS in the presence of capsule, are functional (i.e., induce complement activation and phagocytosis), and are not hindered by structural RPS variation on natively expressed RPS. In conclusion, this conserved RPS motif warrants further investigation as a potential target for the development of a broad-spectrum vaccine against the diverse zoonotic pathogen *S. suis*.

## DISCUSSION

A broadly protective *S. suis* vaccine would not only prevent disease in pigs but also reduce antibiotic use and lower the risk of human infections through zoonotic transmission. Here, we provide an initial molecular dissection of *S. suis* RPS, defining the RPS biosynthesis gene cluster and elucidating the glycosyl linkage patterns of the two structural RPS variants expressed by pathogenic *S. suis* lineages*.* We identified a glycan motif shared by the RPS variants that was immunogenic in piglets and elicited functional antibodies that recognized a broad panel of genetically diverse strains in the presence of capsule. Our findings warrant further exploration on the use of RPS in *S. suis* vaccines.

We demonstrated that SrpP is essential for side-chain biosynthesis, which is in line with the function of the GacI homolog in *S. pyogenes* (*22*, *29*). In *S. pyogenes*, GacJ forms a complex with GacI and significantly stimulates its catalytic activity (*29*). As SrpQ contains a similar functional domain as GacJ, we infer that SrpP is a GlcNAc-P-Und producer, which initiates the side-chain biosynthesis supported by SrpQ. There is also an overlap between the GAC and SCC side chains and the *S. suis* RPS side chain with regard to function. First of all, we demonstrated that the side chain of RPS is crucial for *S. suis* growth and morphology as well as resistance to lysozyme, consistent with findings in *S. pyogenes* and *S. mutans* (*20*, *22*, *27*, *40*). Moreover, the GAC side chain is considered to be a virulence determinant in multiple *S. pyogenes emm* types (*22*, *41*). Similarly in *S. suis*, *srpP* was identified to be essential for bacterial survival in the blood, brain, and cerebrospinal fluid of pigs in a transposon insertion mutagenesis assay (*42*) and up-regulated when *S. suis* is cultured in pig blood compared to Todd-Hewitt broth (*43*). In addition, deletion of *srpP* significantly decreased biofilm formation (*44*). Together, the RPS side chain is likely to be more broadly involved in *S. suis* virulence and pathogenesis.

We successfully conjugated the conserved RPS motif to a carrier protein using click chemistry and used this construct to immunize piglets. Although all piglets showed an IgG response to the carrier protein on day 28, only 7 (58%) of 12 piglets developed IgG responses against the immunizing glycan. Since the immune system of piglets is not fully developed until about 4 weeks of age (*45*–*47*), the first immunization at 3 weeks of age may partially explain this incomplete response. In addition, we only attempted a single immunizing strategy and adjuvant, which may not have been optimal for inducing glycan-specific antibodies in piglets (*8*). These findings are in line with results from a piglet immunization study using conjugated capsule [CPS type 2 (CPS-2)] plus adjuvant in an immunization schedule similar to ours. In this study, only 40% of the piglets generated a CPS-2–specific IgG response in the final assessment (*13*). For implementation of a *S. suis* vaccine in practice, the vaccine would actually be applied to sow, which would then transfer the IgG antibodies to the piglets (*48*). Therefore, future studies should explore alternative immunization conditions and adjuvants to improve glycan-reactive IgG responses in pigs.

The IgG antibodies in piglets that responded to vaccination did not only bind to the core glycan motif that was used for immunization but also recognized the two mature RPS glycan variants expressed by pathogenic *S. suis* strains. Consequently, we were able to demonstrate broad reactivity to *S. suis* strains from various genetic backgrounds. Although the capsule impaired IgG access to RPS expressed on the bacterial surface, the RPS-reactive antibodies promoted robust C3b deposition on the surface of encapsulated *S. suis* strains, implying an important role in classical complement pathway–mediated opsonophagocytosis. Similar observations were made by Baums *et al.* (*49*), where the authors showed that the levels of *S. suis*–opsonizing IgG antibodies in serum postvaccination with a *S. suis* bacterin correlated with opsonophagocytic killing and protection against challenge with a homologous strain. The opsonizing antibodies were directed against surface components other than CPS. In addition to complement activation, the RPS-reactive IgG antibodies alone were potent opsonins as demonstrated by their ability to enhance opsonophagocytosis of RPS-coated beads. Overall, our data demonstrate the accessibility of and binding to the native RPS structural variants by vaccine-induced antibodies in the presence of capsule expression as well as functionality of RPS-induced IgG. Nevertheless, further studies are needed to clarify the protective role of the vaccine-induced RPS-specific antibodies, including the ability to enhance opsonophagocytic killing of *S. suis* and protection in an immunization-challenge experiment.

The commensal *S. suis* lineages showed clearly different genetic architecture and RPS biosynthesis gene sequences compared to pathogenic lineages. This is of interest, since this observation could imply that vaccination with RPS of pathogenic *S. suis* lineages may not affect the commensal nonpathogenic *S. suis* population. If true, this would be beneficial for pig health and reduce the problem of pathogenic strain replacement.

In summary, we identified and characterized the biosynthetic gene cluster and glycosyl linkage patterns of two structural RPS variants from pathogenic *S. suis*. In addition, we demonstrated that the RPS side chain is important for *S. suis* growth and morphology. The two RPS variants shared a core glycan domain that elicited functional antibodies that were reactive against a broad range of pathogenic *S. suis* strains, which merits future exploration as a component of universal vaccines against the zoonotic pathogen *S. suis*.

## MATERIALS AND METHODS

### Identification of *S. suis* RPS biosynthesis gene cluster

The genome sequence of *S. suis* strain P1/7 (*50*) (National Center for Biotechnology Information accession: NC_012925.1) was used as the reference. Orthologs of RPS genes were identified by BLAST (https://blast.ncbi.nlm.nih.gov/Blast.cgi) (*51*). Protein annotation was performed using UniProt (https://uniprot.org/) (*52*), InterPro (https://ebi.ac.uk/interpro/) (*53*), or Pfam (integrated into InterPro in 2022) (*54*) to identify functional domains. In addition, glycosyltransferases were identified using CAZy (http://www.cazy.org/) (*55*), which is a database for carbohydrate-active enzymes. Genes were visualized by ggplot2 (version 3.3.2) (*56*) and gggenes (version 0.4.0) (*57*) in R (version 4.0.3) (*58*, *59*). RPS gene cluster alignments were generated with Clinker (version 0.0.23) (*60*), and GC content of RPS cluster was visualized by Artemis (version 18.1.0) (*61*).

### Collection of *S. suis* genomes

A database containing 1719 publicly available *S. suis* genomes was constructed previously (*28*). Briefly, 1800 *S. suis* genomes along with associated metadata were collected from public databases. Genome quality was assessed by Quality Assessment Tool for Genome Assemblies (version 5.0.2) (*62*), any genome with more than 500 contigs, a N50 lower than 10 kbp, a genome size outside 1.6 to 3.0 Mbp, a GC content outside 40.0 to 42.5%, or more than 50 uncalled bases (Ns)/100 kbp was excluded. Following these quality control measures, 1719 genomes were retained for analysis in this study.

### Phylogenetic analysis

*S. suis* genomes were annotated by Prokka (version 1.14.0) (*63*), and the results were used as the input for Roary (version 3.12.0) (*64*) to identify the core genome. Core genome sequences were subsequently aligned by Multiple Alignment using Fast Fourier Transform (MAFFT) (version 7.307) (*65*), and sites without single-nucleotide polymorphisms (SNPs) were removed by SNP-sites (version 2.5.1) (*66*). The resulting alignment was used to construct a maximum-likelihood phylogenetic tree in IQ-TREE (version 1.6.6) (*67*) with 1000 bootstrap replicates using the GTR+F+I+G4 model (*68*, *69*). The final tree was visualized and annotated by Interactive Tree of Life (iTOL) (*70*).

### Conservation analysis of rhamnose and RPS genes

Putative dTDP-rhamnose and RPS biosynthesis genes were screened across the 1719 genomes by ABRicate (https://github.com/tseemann/abricate) with an initial threshold of 60% coverage and 80% identity. The output was further selected using a more stringent threshold of 90% coverage and 80% identity. Gene conservation was assessed by multiplying coverage and identity and was visualized as heatmaps by iTOL (*70*), together with the phylogenetic tree, ST, CPS type (CPS, serotype), host, and geographical location. Only STs consisting of more than 15 strains and CPS types consisting of more than 25 strains were highlighted.

### Nucleotide variation analysis

The nucleotide sequence of P1/7 whole RPS cluster was screened across the collected genomes by ABRicate (https://github.com/tseemann/abricate, version 1.0.1) using cutoff values of 90% coverage and 80% identity to identify the coordinates of homologous RPS cluster in each genome. These coordinates were used to extract the whole RPS sequences by extract_genes_ABRicate (https://doi.org/10.5281/zenodo.18218680), resulting in 1350 RPS identified sequences. A more stringent selection was then applied using cutoff values of 95% coverage and 80% identity. Multiple sequence alignment of the selected whole RPS cluster sequences of isolates in pathogenic lineages was made by MAFFT (version 7.471) (*65*) after removing sequence gaps by BioEdit (version 7.0.9.0) (*71*). The aligned sequences were entered into ClustalX (version 2.1) (*72*) to calculate the “column scores,” showing nucleotide site conservation. The variation score was calculated as (100 − column score) and visualized by ggplot2 (version 3.3.2) (*56*) in R (version 4.0.3) (*58*, *59*).

### Bacterial strains and culture conditions

The principal strains analyzed in this study were *S. suis* strain S10 (ST-1, CPS-2) (*73*), strain 861160 (ST-20, CPS-2) (*74*), and their capsular-deficient mutants S10 ΔCPS (*75*) and 861160 ΔCPS. In addition, *S. suis* strains representing various CPS types and STs were included in the IgG binding assay. A complete list of strains used in this study (*50*, *73*–*77*) is provided in table S4. *S. suis* strains were cultured in Todd-Hewitt broth (Oxoid) supplemented with 0.5% yeast extract (Bacto) (THY) at 37°C with gentle shaking (120 rpm). *Escherichia coli* strains for cloning purposes were cultured in Luria-Bertani media at 37°C with shaking at 200 rpm. When required, antibiotics were added at the following concentrations: chloramphenicol at 10 μg/ml for *E. coli*, kanamycin at 200 μg/ml for *S. suis*, spectinomycin at 100 μg/ml for *S. suis*, and chloramphenicol at 5 μg/ml for *S. suis*.

### Genetic manipulation of *S. suis*

All *S. suis* mutants were constructed by homologous recombination using competence-inducing peptides (*78*). For construction of the 861160 ΔCPS mutant, the fragment for inactivating the *cpsEF* genes was amplified from S10 ΔCPS mutant (*75*) and transformed to 861160 WT strain. To construct *srp* gene deletion mutants, precise in-frame allelic replacement was performed. Briefly, the upstream and downstream regions of targeted *srp* genes were amplified from genomic DNA and ligated to the kanamycin-resistant Janus cassette (*79*) using overlapping polymerase chain reaction (PCR; Phusion Hot Start II DNA Polymerase, Thermo Fisher Scientific, F549S) to construct the knockout fragment, which was subsequently transformed to *S. suis* strains. All mutants were confirmed using PCR and whole-genome sequencing.

Genetic complementation of *srp* gene deletion mutants was performed using the plasmid vector pDC123 (*80*). The *srp* genes were amplified from genomic DNA using primers containing BglII and BamHI restriction sites. Digested PCR products were subsequently ligated to digested plasmid vector using FastDigest Restriction Enzymes (Thermo Fisher Scientific, FD0083 and FD0054) and T4 DNA Ligase (Roche, 10716359001). The ligation products (recombinant plasmid) were verified by PCR and propagated in *E. coli* strains to gain sufficient quantities for complementation. The plasmids were then introduced into *S. suis* mutants using competence-inducing peptides (*78*). The successful complementation of the gene was confirmed by colony PCR and Sanger sequencing. A complete list of plasmids and primers used in this study is provided in tables S4 and S5, respectively.

### Cell wall isolation

Overnight bacterial cultures were diluted 1:20 in fresh THY broth and grown to late exponential phase [optical density at 600 nm (OD_600_) ≈ 0.7]. Bacteria were harvested by centrifugation in 4°C, and cell walls were isolated using the SDS-boiling procedure, as described before (*20*, *81*). Isolated cell wall samples were lyophilized and stored at −20°C until further analysis (*30*).

### RPS isolation and purification

*S. suis* RPS was released from the cell wall by mild acid hydrolysis after chemical *N*-acetylation, as described previously (*30*). Isolated RPS was purified by size exclusion chromatography on a BioGel P150 (Bio-Rad, 81303) column equilibrated in 0.2 N sodium acetate (NaOAc) (pH 3.7) and 0.15 M sodium chloride (NaCl). If necessary, the fractions were further purified by ion-exchange chromatography on DEAE-Toyopearl column and eluted with NaCl gradients (0 to 0.5 M) (*30*). Total rhamnose and hexose contents were estimated by a modified anthrone assay, as described before (*20*, *30*).

### Phosphate assay

During chromatography, total phosphate content of each fraction was determined by a malachite green method, as described previously (*30*). The total phosphate content of purified RPS was determined by a Malachite green phosphate assay kit (Cayman Chemical, 10009325) after hydrolysis and enzymolysis. In detail, purified RPS (equivalent to 700 nmol of rhamnose) was heated in 2 N hydrochloric acid (HCl) at 100°C for 2 hours in a total volume of 250 μl. The solution was then cooled on ice and neutralized by sodium hydroxide (NaOH) in the presence of 62.5 mM Hepes (pH 7.5). The pH after neutralization was checked by indicator strips. Following this, 50 μl of the acid hydrolyzed sample was incubated overnight at 37°C with 1 U of alkaline phosphatase (Thermo Fisher Scientific, 78390), 10 μl of 10× alkaline phosphatase buffer, and 1 μl of 0.1 M magnesium chloride (MgCl_2_)_,_ in a total volume of 100 μl, with continuous rotation. Released phosphate was quantified using the Malachite green phosphate assay kit according to the manufacturer’s instructions.

### Lectin or antibody binding to whole bacteria

Bacterial overnight culture was diluted 1:20 in fresh THY and incubated to mid-exponential phase (OD_600_ = 0.4 to 0.6) at 37°C with gentle shaking. The culture was harvested by centrifugation at 4000*g* for 10 min. The bacterial pellet was resuspended in phosphate-buffered saline supplemented with 0.1% bovine serum albumin (PBS-BSA buffer) and stored at −20°C until further analysis. For analysis, bacterial suspensions were thawed on ice and further diluted to OD_600_ = 0.048 in PBS-BSA buffer. Subsequently, 12.5 μl of bacterial suspension was incubated in a 96-well V-bottom plate with the same volume of pig serum or fluorescein-labeled lectin (Vector Laboratories, FLK-2100 and FL-1021S-5). The mixture was incubated for 20 min at 4°C in the dark, followed by washing using 125 μl of PBS-BSA buffer and centrifugation at 4000*g* for 10 min. For antibody binding, secondary staining was performed by resuspending the washed bacterial pellet in 25 μl of goat anti-pig IgG (Fc):fluorescein isothiocyanate (FITC) (10 μg/ml; Bio-Rad AAI41F) and incubating for 20 min at 4°C in the dark, followed by washing once with PBS-BSA buffer. Bacteria were further stained in 50 μl of Hoechst 33342 (5 μg/ml; Invitrogen H1399) in PBS for 1 hour in the dark at room temperature, followed by washing with PBS-BSA buffer and resuspended in 100 μl of 1% formaldehyde (Sigma-Aldrich, 252549) in PBS before acquisition on flow cytometer (CytoFLEX, Beckman Coulter Life Sciences). The final concentrations of lectins or sera after mixing with bacteria were as follows: pig serum at 1:100 dilution, *R. communis* agglutinin I (RCA I or RCA_120_) at 5 μg/ml, SBA at 5 μg/ml, and sWGA at 0.25 μg/ml.

### RPS biotinylation and coating to beads

RPS biotinylation was conducted by reductive amination using biotin-amine (AxisPharm, AP10507). Purified RPS was incubated with biotin-amine and sodium cyanoborohydride (NaCNBH_3_; Sigma-Aldrich, 156159) at a molar ratio of 1:600:2500 in 80 mM NaOAc (pH 5.5). Dimethyl sulfoxide was used to increase the solubility of biotin-amine. The reaction was left at room temperature overnight in the dark. The biotinylated RPS was purified by washing with Milli-Q water at least eight times using centrifugal filter units [Amicon Ultra Centrifugal Filter, 3-kDa molecular weight cut-off (MWCO); Millipore, UFC5003] as per the manufacturer’s recommendations (40° fixed angle rotor, 14,000*g*, room temperature).

Biotinylated RPS was coated to streptavidin beads (Dynabeads M-280; Invitrogen, 11205D) by adding 5 × 10^7^ beads (in 20 μl of PBS) to 60 μl of 0.2 mM RPS and incubating at room temperature for 15 min with shaking. The coated beads were washed three times using PBS and resuspended in 1.25 ml of PBS-BSA buffer supplemented with 0.05% Tween 20 (PBS-BSA-T_20_ buffer). The coating of RPS beads was validated by lectin binding assay and analyzed by flow cytometry (BD FACSCanto II, BD Biosciences).

### Lectin or antibody binding to RPS-coated beads

An amount of 1 × 10^5^ RPS-coated beads or uncoated beads in 12.5 μl of PBS-BSA-T_20_ buffer was incubated with the same volume of pig serum or fluorescein-labeled lectin (Vector Laboratories) in a 96-well U-bottom plate at 4°C for 20 min in the dark with shaking. The beads were washed once using a plate magnet (Invitrogen, 12331D). For antibody binding, the secondary staining was performed by resuspending the beads in 25 μl of goat anti-pig IgG (Fc):FITC (10 μg/ml; Bio-Rad, AAI41F) and incubating for 20 min at 4°C in the dark with shaking, followed by washing once using PBS-BSA-T_20_ buffer. The stained beads were resuspended in 100 μl of PBS-BSA-T_20_ buffer and analyzed by flow cytometry (BD FACSCanto II, BD Biosciences or CytoFLEX, Beckman Coulter Life Sciences). The concentrations of lectins or sera after mixing with beads were the same as those used to stain the whole bacteria.

### C3b deposition assay

Overnight cultures of *S. suis* were diluted 1:20 in fresh THY and incubated at 37°C with gentle shaking at 120 rpm until OD_600_ = 0.4 to 0.6, harvested, and cryopreserved at −80°C in the presence of 14% glycerol. 861160 WT RPS-coated Dynabeads M280 (2 × 10^5^) or bacteria (3 × 10^5^, strains S10 and 8067) in RPMI 1640 L-glutamine Hepes (Gibco, 22400089) with 0.05% BSA (assay buffer) were mixed with 4% heat-inactivated pooled test sera and 10% pooled serum from cesarean-derived colostrum-deprived piglets (CDCD serum) as a complement source in a total volume of 25 μl. The mixture was incubated for 45 min at 37°C with constant shaking (650 rpm). Next, beads or bacteria were washed with ice-cold PBS-BSA buffer, with Tween_20_ for the beads, and stained with FITC-labeled rabbit anti-human C3c antibody (Agilent, F0201). Following fixation with 1% paraformaldehyde, with the addition of DNA staining Hoechst 33342 for the bacterial samples, samples were analyzed on a CytoFLEX flow cytometer.

### Opsonophagocytosis of RPS-coated beads by pig neutrophils

Heparinized blood was collected from 8-week-old pigs from a Dutch high health farm. Fresh porcine neutrophils were isolated using methods described previously (*82*). Briefly, heparin blood was mixed 1:1 with Dulbecco’s PBS without Ca^2+^ and Mg^2+^ (Gibco, 14190144), layered on Ficoll Paque Plus (1.077 g/ml; Cytivia, 17144002) and subsequently centrifuged for 30 min at 400*g* at room temperature. The plasma, peripheral blood mononuclear cells, and Ficoll fractions were removed, and the erythrocytes and polymorphonuclear neutrophils (PMNs) containing pellet were retained. Next, the erythrocytes were removed from the pellet by hypotonic lysis, and the purified PMNs were washed and suspended in assay buffer.

An amount of 5 × 10^5^ FITC-labeled, 861160 WT RPS-coated Dynabeads M280 in assay buffer were incubated with 8 or 2% pooled test sera and 10% inactivated CDCD serum in a total volume of 25 μl. The mixture was incubated for 15 min at 37°C with constant shaking at 650 rpm, followed by addition of 1.5 × 10^5^ porcine neutrophils (in 25 μl; final concentrations of test sera, 4 and 1%), and incubation at 37°C with constant shaking at 650 rpm for 30 min. Phagocytosis was terminated by adding 150 μl of ice-cold PBS with 1.5% paraformaldehyde and incubation at 4°C for 30 min, followed by 30 min at room temperature. The cells were washed and resuspended in PBS before acquisition on a CytoFLEX flow cytometer.

### Flow cytometry data analysis

Data acquired by flow cytometer were analyzed using FlowJo (version 10.10.0). The bacterial population was gated first on the basis of the Hoechst 33342–positive population and then using the forward and side scatterplots. The single bacteria population was further selected using the forward-area and forward-height plot. Bacteria stained by only secondary antibody (no pig serum) in bacteria-antibody binding assay or unstained bacteria in bacteria-lectin binding assays were used as background staining. In contrast, the single bead population was gated on the basis of the forward and side scatterplots. Uncoated beads stained by either lectin or primary serum and secondary antibody were used as background staining. For the neutrophil phagocytosis assay, total neutrophils were gated on the basis of SSC and FITC signal to exclude beads and other cells and debris. Within the total neutrophil population, bead-positive cells were gated on the basis of the FITC signal. The antibody level or lectin binding level was represented by delta geometric mean fluorescence intensity (ΔGMFI), which was calculated by subtracting the GMFI of background staining from the GMFI of tested samples.

### Glycosyl composition and linkage analysis

Purified RPS was reduced in 0.1 M ammonium hydroxide (NH_4_OH; Sigma-Aldrich, 221228) and sodium borohydride (NaBH_4_; 10 mg/ml; Sigma-Aldrich, 213462) at room temperature overnight, followed by neutralization using acetic acid (CH_3_COOH). Reduced RPS was analyzed by combined GC-MS in the Complex Carbohydrate Research Center (University of Georgia, Athens, USA) using trimethylsilylated methyl glycoside derivatization for glycosyl composition and using methylation for linkage determination, as described previously (*83*).

### NMR spectroscopy

RPS from *S. suis* were dissolved in D_2_O (0.55 ml). NMR experiments were conducted in 5–mm–outer diameter NMR tubes on Bruker NMR spectrometers operating at ^1^H frequencies of 400 or 700 MHz at temperatures of 23° or 50°C, respectively, using experiments suitable for resonance assignments of glycans (*35*, *84*). ^1^H NMR chemical shifts were referenced to internal sodium 3-trimethylsilyl-(2,2,3,3-^2^H_4_)-propanoate (δ_H_ 0.0), ^13^C chemical shifts were referenced to external dioxane in D_2_O (δ_C_ 67.4), and ^31^P chemical shifts were referenced to external 2% H_3_PO_4_ in D_2_O (δ_P_ 0.0). Acquired NMR data were processed and analyzed using the TopSpin software from Bruker.

### Growth curve

Overnight bacterial cultures were diluted in fresh THY without antibiotics to OD_600_ ≈ 0.4, subsequently further diluted 20-fold and aliquoted to two test tubes, 5 ml per tube, and recorded the OD_600_ values as time = 0 hours. All tubes were incubated at 37°C with gentle shaking. The OD_600_ value of each tube was measured every 30 min. An extra tube filled with 5 ml of fresh THY was used as blank control. Growth curves were made using the average OD_600_ of the aliquots after subtraction of the blank control.

### Chain length quantification

Stationary phase bacteria from overnight cultures in THY broth were used for Gram staining. Multiple pictures were taken for every strain under 40× microscope objective. Chain length was quantified by counting the pixels of all bacterial chains in every single picture using ImageJ (*85*), until at least 200 chains were analyzed. The number of pixels was transferred to length (in micrometers) and used for further analysis.

### Scanning electron microscopy

Overnight bacterial cultures were diluted 1:10 in fresh THY and incubated to exponential phase (OD_600_ ≈ 0.4) at 37°C with gentle shaking. Four milliliters of bacterial culture was harvested by centrifugation at 4000 rpm for 10 min at room temperature. The pellet was resuspended in 1 ml of fixative [3% glutaraldehyde/3% paraformaldehyde in 0.1 M phosphate buffer (pH 7.4); Electron Microscopy Sciences, 16538-06] and incubated at room temperature for 30 min. Samples were stored at 4°C until analysis.

*S. suis* cultures in fixative were washed with H_2_O, followed by a dehydration with increasing concentrations of alcohol. Samples were incubated with 30, 50, 70, 80, 90, 96, and 100% alcohol for 10 min per step at room temperature. After each step, samples were centrifuged at 5000 rpm for 5 min, and the supernatant was removed. Samples were adhered to glass slides covered with poly-l-lysin and rapidly critically point dried using liquid CO_2_ (Leica Microsystems, CPD300; auto mode, CO_2_ in speed slow, exchange speed of 3 with 14 cycles, and gas out heat slow at speed slow 100%). Subsequently, samples were attached to aluminum stubs (Agar Scientific, AGG301) using carbon stickers (Agar Scientific, G3347) and sputter coated with 6-nm platinum/palladium coating (Leica Microsystems, ACE600). Samples were then imaged using a Zeiss Gemini Sigma 300 FEG scanning electron microscopy at 3 kV using an SE2 detector.

### Transmission electron microscopy

*S. suis* cultures in fixative were washed with H_2_O for 10 min and incubated for 1 hour in osmium tetraoxide (1% in double distilled H_2_O). Samples were then washed with H_2_O for 10 min and dehydrated with increasing concentrations of alcohol (70, 70, 80, 90, 90, 96, 100, and 100% for 15 min per step). After each step, samples were centrifuged at 5000 rpm for 5 min, and the supernatant was removed. Then, samples were impregnated with 1:1 epon (Electron Microscopy Sciences) and propylene oxide for 2 hours followed by centrifuging for 10 min at 7000 rpm and replacing epon/propylene oxide mixture with 100% epon. After incubation overnight, epon was replaced with fresh epon and polymerized at 60°C for 2 days. Samples were sectioned using a diamond knife (Diatome) and ultramicrotome (Leica Microsystems, EM UC7) in 60-nm sections and placed on copper grids followed by poststaining with uranyl acetate (3.5% in ddH_2_O) and lead citrate (Electron Microscopy Sciences, 22410). Samples were then imaged partly using a Talos L120c TEM with 16 M BM-CETA camera and partly using a Tecnai T12 TEM with Xarosa camera.

### Lysozyme resistance assay

Knocking out *srp* genes resulted in *S. suis* chain length changes, affecting colony-forming units. We alternatively used an OD_600_-based strategy to analyze *S. suis* lysozyme resistance. In detail, bacterial overnight cultures were diluted 1:10 in fresh THY and subcultured to mid-exponential phase (OD_600_ = 0.4 to 0.6) at 37°C with gentle shaking. All subcultures were adjusted to OD_600_ = 0.4. To harvest the bacteria, 5 ml of OD_600_ = 0.4 culture was centrifuged at 4000*g* for 5 min at room temperature and washed once using PBS. The bacterial pellet was resuspended in 1 ml of PBS. Subsequently, 100 μl of bacteria suspension was incubated together with lysozyme from chicken egg white (Sigma-Aldrich, L6876) in a 96-well flat-bottom plate. The OD_600_ value was measured immediately to check the input of the bacteria. The plate was incubated at 37°C for 1.5 hours with gentle shaking, and the OD_600_ value was measured afterward. Bacteria incubated with PBS [lysozyme (0 μg/ml)] served as controls. The survival factor was calculated by dividing the OD_600_ value of the tested samples by the OD_600_ value of the controls.

### Preparation of glycan-protein conjugates

861160 Δ*srpL* RPS was used to prepare the glycoconjugate. RPS was extracted from the cell wall by mild acid hydrolysis and then purified by size exclusion chromatography, following the method described above. The purified RPS was derivatized at their reducing end with alkyne-hydrazide (Lumiprobe, 41770) through reductive amination, using a molar ratio of RPS:hydrazide:NaCNBH_3_ = 1:300:1000. This reaction was conducted in 100 mM NaOAc at pH 4.5, at room temperature, overnight with gentle shaking. The alkyne-derivatized RPS was subsequently purified by ultracentrifugation, through 25 cycles of washing with Milli-Q water (with each cycle involving approximately a twofold dilution), using centrifugal filter units (Amicon Ultra Centrifugal Filter, 3-kDa MWCO; Millipore, UFC5003) as per the manufacturer’s instructions (40° fixed angle rotor, 14,000*g*, room temperature).

The alkyne-derivatized RPS was then conjugated to CRM_197_-azide (CRM_197_-N_3_) using click chemistry (CuAAC) (*39*). Conjugation-ready CRM_197_-N_3_ was ordered from Fina Biosolutions, prederivatized with approximately 15 azide groups for reaction with alkyne groups (https://finabio.net/product/crm-azide/). The click reaction was carried out with final concentrations of copper sulfate (Cu_2_SO_4_) at 1 mM, sodium ascorbate at 10 mM, tris(benzyltriazolylmethyl)amine at 1 mM, and aminoguanidine at 10 mM in 30 mM Hepes buffer at pH 8.0. Different ratios of CRM_197_-N_3_ and RPS-alkyne were tested to determine the optimal ratio, which was analyzed via SDS–polyacrylamide gel electrophoresis (SDS-PAGE). The final chosen molar ratio of azide:alkyne was 1:0.7, corresponding to CRM_197_:RPS = 1:10.5, for glycoconjugate synthesis. The reaction was proceeded at room temperature for 1 hour, after which a sample was taken. The sample reaction was stopped by adding Laemmli sample buffer supplemented with β-mercaptoethanol (Bio-Rad, 1610747) and analyzed by SDS-PAGE (fig. S7A). The conjugate was purified by ultracentrifugation, with 25 cycles of washing in PBS, using centrifugal filter units (Amicon Ultra Centrifugal Filter, 30-kDa MWCO; Millipore UFC8030) as per the manufacturer’s recommendations (swinging bucket rotor, 4000*g*, room temperature), followed by sterile filtration through 0.22-μm filters. The filter was washed twice with PBS to maximize recovery. Protein and glycan concentrations were measured using the BCA protein assay kit (Pierce BCA Protein Assay Kits, Thermo Fisher Scientific, 23227) and a modified anthrone assay, respectively.

### Pig immunization and serum collection

CRM_197_-RPS conjugate was adjuvanted with X-Solve, an oil in water emulsion consisting of a 5 to 1 (v/v) blend of a paraffin-based micro-emulsion and a vitamin E acetate-based nano-emulsion, mixed in a volume ratio of 1:1 (*48*). Twenty-four 3-week-old piglets (Duroc and Yorkshire, both sexes) were divided into two groups: a vaccine group and an unvaccinated control group. All piglets were born and housed on the same farm during the study, with litters evenly distributed across treatment groups. Piglets were immunized intramuscularly twice at a 2-week interval (days 0 and 14, corresponding to 3 weeks and 5 weeks of age, respectively) with 2 ml of formulation (corresponding to 109 μg of carrier protein). Piglets were weaned 3 days after the first vaccination and then housed in groups. Blood samples were collected on study days 0, 14, and 28 to determine serum antibody levels.

Total serum IgG against RPS and whole bacteria was measured by flow cytometry, and CDCD serum was used as negative control. In contrast, total serum IgG against the carrier protein was determined by enzyme-linked immunosorbent assay.

### Ethics statements of animal experiment

The Dutch Central Authority for Scientific Procedures on Animals and the Animal Welfare Bodies of MSD Animal Health and Wageningen University and Research approved of the described animal studies (Act on Animal Experimentation permit numbers AVD22100202114853 and AVD40100202317645, respectively). All researchers performing experimental procedures were qualified to handle the animals in accordance with the Experiments on Animals Act. Housing and management conditions were in line with the EU Directive 2010/63/EU on the protection of animals used for scientific purposes. The health and behavior of the pigs were monitored daily by certified animal caretakers.

### Enzyme-linked immunosorbent assay

Nunc MaxiSorp plates (Thermo Fisher Scientific) were coated overnight at 4°C with CRM_197_ carrier protein (0.57 μg/ml) in carbonate buffer. After coating, the plates were blocked with 1% BSA in 0.04 M PBS for 1 hour at 37°C. After blocking, the plates were washed three times. Serum was added and incubated for 1 hour at 37°C. For the detection of total IgG, plates were incubated with peroxidase-labeled goat anti-swine IgG (0.1 μg/ml; heavy plus light chain, SeraCare, 5220-0363) for 1 hour at 37°C. After each incubation step, the plates were washed. The plates were developed with trimethylboron as substrate, and absorbances were measured at 450 nm.

Every sample was measured in a singular series of eight threefold dilutions starting with 1:160. A pool of control group sera on day 28 served as background control at 1:160. The antibody titers were defined as the interpolated reciprocal of the dilution corresponding to the OD value equal to twice the average background absorbance and are expressed as log_2_ titer.

### Statistical analysis

All data were analyzed and visualized using GraphPad Prism (version 10.2.0). The specific statistical methods are detailed in the figure captions. A *P* value of ≤ 0.05 was considered statistically significant.
